# Adjunction of a fish oil emulsion to cytarabine and daunorubicin induction chemotherapy in high-risk AML

**DOI:** 10.1038/s41598-022-13626-y

**Published:** 2022-06-13

**Authors:** Emmanuel Gyan, Arnaud Pigneux, Mathilde Hunault, Pierre Peterlin, Martin Carré, Jacques-Olivier Bay, Caroline Bonmati, Maria-Pilar Gallego-Hernanz, Bruno Lioure, Philippe Bertrand, Nicolas Vallet, David Ternant, François Darrouzain, Frédéric Picou, Marie-Christine Béné, Christian Récher, Olivier Hérault

**Affiliations:** 1 Service d’Hématologie et Thérapie Cellulaire, Centre Hospitalier Universitaire de Tours, Hôpital Bretonneau, Bâtiment Kaplan, 2, boulevard Tonnellé, 37044 Tours Cedex 09, France; 2 ERL CNRS 7001, Leukemic Niche and Redox Metabolism (LNOx), Faculté de Médecine, Université de Tours, Tours, France; 3 Centre d’Investigation Clinique, INSERM U1415, Centre Hospitalier Universitaire, Tours, France; 4 Clinique d’Hématologie, Université de Bordeaux, Hôpital Haut-Levêque, Pessac, France; 5 Service des Maladies du Sang, FHU GOAL, CRCINA, INSERM Angers, Centre Hospitalier Universitaire, Tours, France; 6 Service d’Hématologie, Centre Hospitalier Universitaire, Nantes, France; 7 Service d’Hématologie, Centre Hospitalier Universitaire, Grenoble, France; 8 Service d’Hématologie, Centre Hospitalier Universitaire, Clermont-Ferrand, France; 9 Service d’Hématologie, Centre Hospitalier Universitaire, Nancy, France; 10 Service d’Hématologie, Centre Hospitalier Universitaire, Poitiers, France; 11 Service d’Hématologie, Centre Hospitalier Universitaire, Strasbourg, France; 12 Laboratoire de Biostatistiques, Faculté de Médecine, Université de Tours, Tours, France; 13 Laboratoire de Pharmacologie-Toxicologie, Centre Hospitalier Universitaire, Tours, France; 14 Laboratoire d’Hématologie, Centre Hospitalier Universitaire, Nantes, France; 15 Service d’Hématologie, Institut Universitaire de Cancérologie de Toulouse, Toulouse, France; 16 Service d’Hématologie Biologique, FHU GOAL, Centre Hospitalier Universitaire de Tours, Hôpital Bretonneau, Bâtiment B2A, 2, boulevard Tonnellé, 37044 Tours Cedex 09, France

**Keywords:** Acute myeloid leukaemia, Leukaemia

## Abstract

The treatment of acute myeloid leukemia (AML) with unfavorable cytogenetics treatment remains a challenge. We previously established that ex vivo exposure of AML blasts to eicosapentaenoic acid (EPA), docosahexaenoic acid (DHA), or fish oil emulsion (FO) induces Nrf2 pathway activation, metabolic switch, and cell death. The FILO group launched a pilot clinical study to evaluate the feasibility, safety, and efficacy of the adjunction of a commercial FO emulsion to 3 + 7 in untreated AML with unfavorable cytogenetics. The primary objective was complete response (CR). Thirty patients were included. FO administration raised the plasma levels of eicosapentaenoic (EPA) and docosahexaenoic (DHA) acids (*p* < 0.001). The pharmacokinetics of cytarabine and daunorubicin were unaffected. A historical comparison to the LAM2001 trial (Lioure et al. Blood 2012) found a higher frequency of grade 3 serious adverse events, with no drug-related unexpected toxicity. The CR rate was 77%, and the partial response (PR) 10%, not significantly superior to that of the previous study (CR 72%, PR 1%). RT-qPCR analysis of Nrf2 target genes and antioxidant enzymes did not show a significant in vivo response. Overall, FO emulsion adjunction to 3 + 7 is feasible. An improvement in CR was not shown in this cohort of high-risk patients. The present data does not support the use of FO in adjunction with 3 + 7 in high-risk AML patients.

ClinicalTrials.gov identifier: NCT01999413.

## Introduction

Acute myeloid leukemias (AML) are a heterogeneous group of hematological malignancies for which therapy is stratified according to cytogenetic and molecular prognostic factors. AML with high-risk cytogenetics leads to a dismal prognosis, is often refractory to induction therapy, and often relapses, even after allogeneic bone marrow transplantation. Hence, it is an unmet medical need. Improving the efficacy of treatment and optimizing the benefit-risk ratio in AML is the subject of intense clinical research.

Polyunsaturated long-chain n-3 fatty acids (n-3 PUFAs) have antitumoral properties that have been demonstrated in various in vitro solid tumor models, such as breast cancer, prostate cancer, and ovarian cancer cell lines. Animal models confirmed an antitumoral effect, with the induction of apoptosis and cell cycle arrest in murine autochthonous colon cancer and mammary rat breast cancer^1^. Among hematological malignancies, the same properties have been described for lymphoid and myeloid cell lines^2–5^. Among AML cell lines and primary blasts, there is evidence that n-3 PUFAs induce apoptotic cell death and a mitochondrial metabolic switch from oxidative phosphorylation to glycolysis, together with activation of the oxidative-stress related NRF2 pathway that is not sufficient to prevent cell death^6^ In human cancer, n-3 PUFAs have been safely administered to women with metastatic breast cancer^7^. A phase II trial evaluating oral supplementation with FO capsules concomitantly with FEC100 chemotherapy showed an association between high n-3 PUFA intake and better outcome^8^.

Fish oil (FO) emulsion (OMEGAVEN®, Fresenius Kabi, Bad Homburg, Germany) contains 1.25–2.82 g eicosapentaenoic acid (EPA) and 1.44–3.09 g docosahexaenoic acid (DHA)/100 mL (manufacturer’s specifications). It is authorized registered as parenteral nutrition supplementation for intravenous (IV) infusion with long chain omega-3-fatty acids. FO supplementation, mostly by oral route, has been associated with an improved nutritional status in patients treated for diverse cancer types^9,10^. In the setting of hematologic malignancies, an increase in EPA and DHA plasma levels has been associated with an improved outcome in a prospective comparative study on 22 patients^11^ Oral supplementation with FO capsules in children treated with methotrexate has been associated with improved liver function^12^. There is evidence of benefit and safety of FO in critically ill patients, such as a reduction of the hospital length of stay after abdominal surgery^13^, and a reduced length of stay in intensive care units for patients with sepsis treated with FO^14^. These data suggest that a high dose of FO can be safely administered to vulnerable patients.

We hypothesized that FO could be safely administered to patients newly diagnosed with high-risk AML, without compromising daunorubicin and cytarabine pharmacokinetics, and that its adjunction to standard 3 + 7 may improve the complete remission rate of induction therapy. We thus set up a feasibility phase II study to evaluate the safety and efficacy of the adjunction of a FO lipid emulsion to daunorubicin and cytarabine induction chemotherapy in untreated AML patients with adverse cytogenetics.

## Patients and methods

### Study design

**Figure 1:**
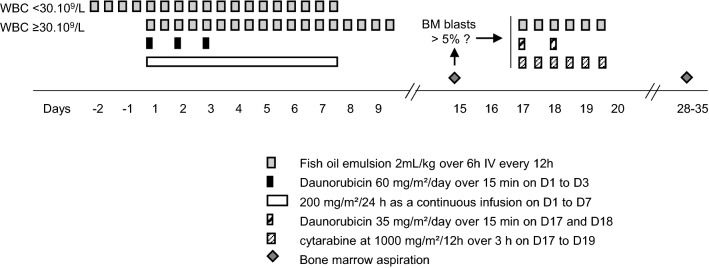
FAMYLY study design. Patients eligible for the study were treated with fish oil emulsion, daunorubicin and cytarabine at the indicated doses. If the baseline white blood count (WBC) was below 30 G/L, patients received 48 of fish oil before the start of chemotherapy. If the WBC was above or equal to 30 G/L, all treatments were started on the same day. An evaluation bone marrow (BM) evaluation was performed on D15. If the BM blasts were higher than 5%, a second induction was provided at the indicated doses.

The single-arm phase II FAMYLY (Fatty acids in Acute MYeloid Leukemia of Younger patients) study design was derived from the previous GOELAMS/FILO LAM2001 study which randomized daunorubicin-based versus idarubicin-based induction chemotherapy^15^ Treatment consisted of daunorubicin at 60 mg/m^2^/day over 15 min on D1 to D3, concomitantly with cytarabine at 200 mg/m^2^/24 h as a continuous infusion on D1 to D7. Fish oil (OMEGAVEN®) was administered at 2 mL/kg over 6 h bid for 9 days, starting on D1 if the white blood cell (WBC) count was ≥ 30 G/L or 48 h before the start of chemotherapy if the WBC was < 30 G/L (Fig. 1). The dose and duration of FO were chosen according to previous experience of IV administration of FO in humans with 350,400 mL/d for 5–9 days, allowing for plasmatic DHA concentrations of 30–60 µM^16,17^ which have a biologic activity in AML cells in vitro^6^. A second induction course was administered if D15 bone marrow blasts were > 5%. It consisted of intravenous (1) daunorubicin at 35 mg/m^2^/day over 15 min on D17 and D18, (2) cytarabine at 1000 mg/m^2^/12 h over 3 h on D17 to D19, and (3) OMEGAVEN at 2 mL/kg/12 h over 6 h on D17 to D19.

The primary objective was the complete response (CR) rate after induction, defined as the presence of < 5% blasts in the bone marrow. The secondary objectives were the tolerance of the addition of FO to induction chemotherapy, blast fusion, and the pharmacokinetics (PK) of n-3 PUFAs, daunorubicin, and cytarabine.

### Study populations

Eligible patients for the FAMYLY study were 18–61 years old and diagnosed with untreated AML (≥ 20% blasts) with high-risk cytogenetics, defined as − 5, 5q abnormalities, − 7, 7q abnormalities, t(6;9), 11q23 abnormalities excluding t(9;11), 3q abnormalities, and a complex karyotype with three or more abnormalities^15^. Adequate liver, heart, and kidney function were required, as well as an ECOG status ≤ 2 and having signed an informed consent form. Patients with history of aplastic anemia, bone marrow transplantation, active infection, CNS involvement, or previous total body irradiation were excluded, as well as patients with a normal or favorable cytogenetic abnormality, such as t(15;17), inv(16), or t(8;21). Hydroxyurea was allowed to wait for the cytogenetic results. The study was amended during accrual to allow for the inclusion of patients younger than 65 years, and for secondary AML (excluding therapy-related AML). Patients from the historical comparator were a subgroup with adverse cytogenetics from the daunorubicin arm of the phase III LAM2001 study^15^. Briefly, the inclusion criteria were non-acute promyelocytic de novo AML younger than 60 years. Adverse cytogenetics were defined as described above.

### Pharmacokinetics

PK blood samples were collected from 10 patients included in the centers volunteering for the PK sub-study. Daunorubicin samples were collected through a peripheral vein distant from the central catheter into lithium heparin tubes within 1 h before and 1 h after each daunorubicin infusion, followed on D3 by sampling at 2, 5, and 10 h post-infusion. The samples were kept on ice and centrifuged at 1300×*g* at 4° within 1 h of the blood draw. Supernatants were immediately aliquoted and frozen at − 80 °C. Cytarabine PK samples were collected at baseline, on D3 (anytime), D7 (anytime), and 45 min and 2, 5, and 10 h after the end of the last cytarabine infusion. Samples were handled as described above. Cytarabine and daunorubicin plasma concentrations were measured by high-performance liquid chromatography (HPLC) as previously described^18^.

PUFA pharmacokinetics samples were collected in EDTA tubes before the first infusion of FO, before the 5th infusion, before the 9th infusion, and on D10 and D28. Tubes were kept on ice and centrifuged at 900×*g* at 4 °C within 1 h of the blood draw and then immediately aliquoted and frozen at − 80 °C. PUFAs were extracted from plasma by methanol transesterification and their concentration measured by gas chromatography, as previously described^19^.

### Antioxidant response in primary cells

Primary mononucleated blood samples were available for a subset of patients at D1, D3, and D5 of treatment and sent to the FILO biobank for freezing in DMSO. Cell RNA was extracted (TRIzol®, ThermoFisher Scientific, Waltham, MA, USA), and 500 ng used for reverse transcription (SuperScript™ VILO™ cDNA Synthesis Kit, ThermoFisher Scientific). Expression of the following genes was quantified on a LightCycler® Instrument II (Roche Life Science, Basel, Switzerland): *HMOX1*, *NQO1*, *SOD1*, *SOD2*, *SOD3*, *CAT*, *TXN*, *TXN2*, *GLRX1*, *GLRX2*, *GLRX2*, *GLRX3*, *GLRX5*, *GPX1*, *GPX2*, *GPX3*, *GPX4*, *GPX7*, *GSR*, *PRDX1*, *PRDX2*, *PRDX2*, *PRDX3*, *PRDX4*, *PRDX5*, *PRDX5*, and *PRDX6* and normalized according to the geometric mean of the expression of the housekeeping genes *ACTB*, *GAPDH*, and *B2M*^20^. Primer sequences are presented in Supplemental Table S1.

### Statistics

Our working hypothesis was that the adjunction of FO was not inferior to chemotherapy alone, without additional toxicity. A minimum CR rate of 50% would thus be possible using the Fleming one-step phase II procedure^21^ considering a CR of 72% with the LAM 2001 daunorubicin-based induction chemotherapy schedule (LAM 2001 D) in the high-risk cytogenetic group with a type I error of 5% and a type II error of 20%. Thus, if 18 of 30 patients or more obtained a CR, a phase III trial would be warranted. Given an expected death rate from induction therapy of 2.4%, the study would be halted and a data safety monitoring board consulted if three toxic deaths occurred. A sensitivity analysis comparing the efficacy and tolerance of the FAMYLY study to that of the LAM 2001 trial was planned. Continuous variables were compared using the Mann–Whitney Wilcoxon test. Dichotomous variables were compared using the chi-2 or Fisher exact test. Statistical analyses were performed using IBM SPSS Statistics 20.0 (IBM, Armonk, NY, USA). Kruskall Wallis tests were performed for gene expression analysis using R software version 3.3.1 (https://www.r-project.org/).

### Ethics

The study was conducted according to the declaration of Helsinki and approved by the Ethics Committee of Tours on February 15, 2013 and by the *Agence Nationale de Sécurité du Médicament* (ANSM) on January 08, 2013. All participants to the study were required to sign an informed consent form prior to any study-related activities. This study was registered on the ClinicalTrials.gov website under the number NCT01999413, on 13/12/2013.

## Results

### Patient characteristics

Between November 13, 2013 and June 17, 2016, 30 patients were included from nine French centers. The median age was 54 years (range 30–64 years) and only three patients were hyperleukocytic. Extramedullary disease was documented for one patient (3%). The FAMYLY cohort was compared to 75 patients with adverse cytogenetics from the daunorubicin arm of the phase III LAM2001 study. The characteristics of patients from both cohorts are presented in Table 1.

**Table 1:** Patient characteristics.

N (%)	FAMYLY	LAM 2001 D
All	30 (100)	75 (100)
Median age (min–max)	54 (30–64)	49 (19–60)
Male sex	18 (60)	46 (61)
WBC > 30 10^9^/L	3 (10)	NR
PS 0–1	26 (87)	64 (85)
Extramedullary disease	1 (3)	NR
Secondary AML	2 (7)	0 (0)
Cytogenetics*		
Complex (≥ 3 abnormalities)	17 (57)	41 (55)
Monosomal	12 (40)	32 (43)
− 17	10 (33)	15 (20)
7q/− 7	8 (27)	30 (40)
11q23 abnormality	8 (27)	17 (23)
3q26 abnormality	7 (23)	7 (9)
5q/− 5	6 (20)	28 (37)
t(6;9)	1 (3)	2 (3)

*NR* not reported, *PS* performance status, *WBC* white blood count.

*The sum exceeds the total because of the presence of multiple abnormalities.

### n-3 PUFA pharmacokinetics

**Figure 2:**
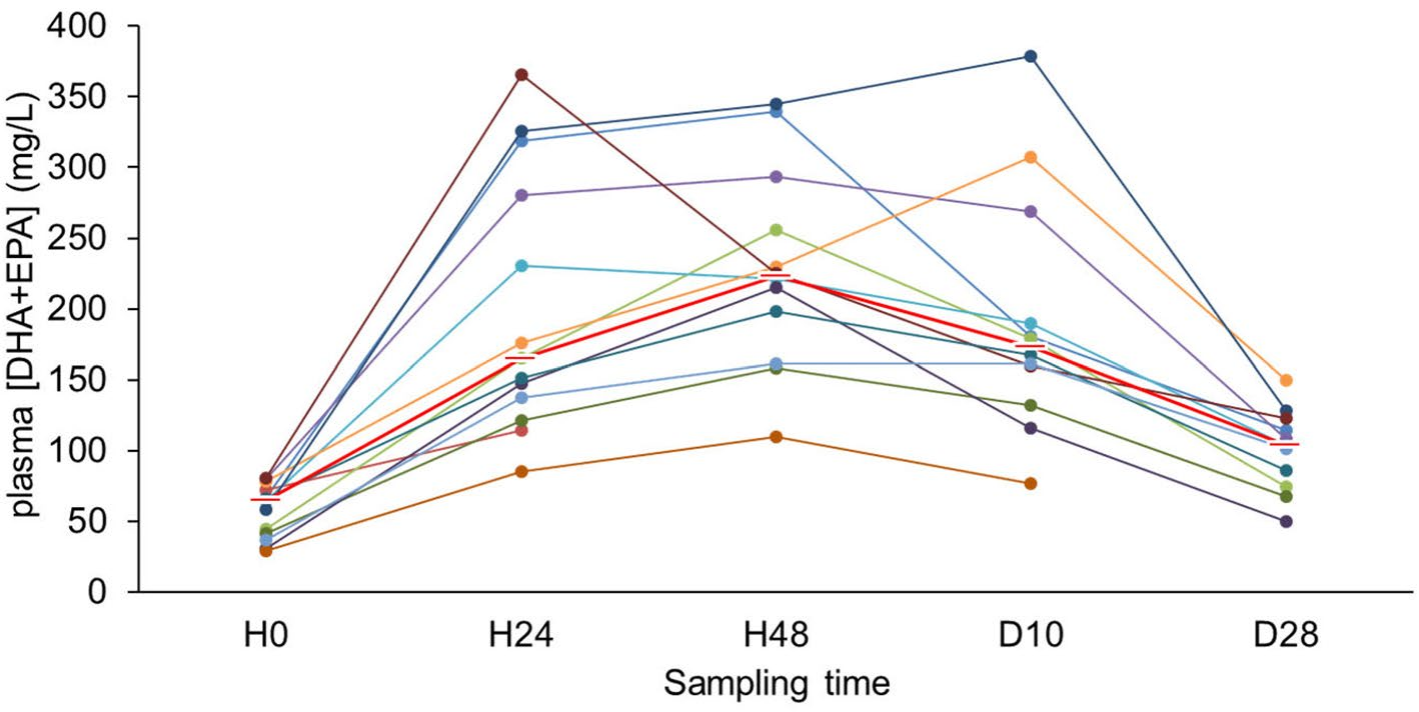
PUFA plasma pharmacokinetics. Plasma samples were drawn at baseline (H0), 24 and 48 h after the start of FO infusion, after the end of infusion (D10), and at disease evaluation (D28–D35). The PUFA composition of plasma was evaluated by gas chromatography. The sum of docosahexaenoic acid (DHA) and eicosapentaenoic (EPA) acid concentration is indicated for each individual patient (colored lines and dots). Means are indicated by a dash for each timepoint.

The sum of the DHA and EPA plasma levels showed a significant increase at 48 h after the start of the FO infusions, with a five-fold increase from 2.1 to 10.5 mg/L (*p* < 0.001, Fig. 2). After stopping FO administration, the plasma [DHA + EPA] concentration decreased to a mean level of 3.8 mg/mL by D28, significantly higher than baseline (*p* = 0.006).

### Effect of FO infusion on WBC counts in vivo

FO infusion as a monotherapy between D-2 and D1 did not reduce the WBC counts of patients without hyperleukocytosis (not shown), whereas the initiation of cytarabine + daunorubicin was associated with the rapid clearance of circulating WBCs.

### Oxidative stress analysis

We observed no significant modulation of *HMOX1*, *NQO1* (Nrf-2 regulated genes), or antioxidant enzymes before or after exposure to IV FO (not shown).

### Tolerance

Twenty-three serious adverse events (SAE) were declared after IV administration of FO in adjunction to 3 + 7 (Table 2). Most were infectious, with no modification of the study drug administration, except for one patient with respiratory failure. A detailed description of the adverse events is shown in Table 3. Of note, no liver impairment nor hemorrhagic events were observed. The mean number of days of thrombocytopenia < 20 10^9^/L was 21 (range [4; 48]) and the number of days of neutropenia < 0.5 10^9^/L was 27 (range [4; 43]). One fatal SAE was recorded but unrelated to FO. We found a higher proportion of grade 3 SAEs, associated with a lower proportion of grade 4 SAEs, when compared to the historical adverse cytogenetic group of the LAM2001 trial (*p* < 0.001).

**Table 2:** Serious adverse events (SAEs) in patients treated in the FAMYLY study compared to the daunorubicin arm of the LAM2001 study in patients with adverse cytogenetics.

	FAMYLY	LAM2001D*	*p* value
**Safety population**	30	78	
**Total declared SAEs, n**	28	30	
Concerned patients, n (%)	17 (56)	25 (32)	0.02
**SAEs, n (%)**			
Grade 3	24 (86)	8 (27)	< 0.001
Grade 4	4 (14)	22 (73)	
Grade 3–4	28 (93)	30 (38)	
Fatal, n (%)	1 (4)	8 (10)	0.44
**Type of SAE, n (%)**			
Infections	16	17	
Documented bacterial infections	2	4	
Documented fungal infections	1	2	
Suspected fungal infections	1		
Without documentation	3	3	
Hepatobiliary events	3	1	
Pain	2		
Cardiac events	1	1	
Vascular events	1	1	
Renal events	1	1	
Respiratory events	1	7	
Gastrointestinal events	1		
Mucositis	1		
Psychiatric disorders	1		
Cutaneous	1	2 (8.0)	
**SAEs related to FO by the investigator, n (%)**	4 (23)	–	–
Fatal	0 (0)		
Infections	3 (66.7)		
Documented bacterial infections	2 (100)^b^		
Gastrointestinal events	1 (33.3)		

*SAE* severe adverse events.

*Daunorubicin arm only.

^a^One case of mucormycosis.

^b^Including one case of aspergillosis and one of invasive candidiasis.

^c^*E. Coli* bacteriemia and *Clostridium difficile* colitis in the same patient.

**Table 3:** Response to induction in the LAM2001D and FAMYLY studies.

	LAM2001D	FAMYLY	*P*
N (%)	75 (100)	30 (100)	
2nd induction	37 (49)	14 (47)	0.81
CR	54 (72)	23 (77)	0.62
PR	1 (1)	3 (10)	0.14
ORR	55 (73)	26 (86)	0.11
Failure	20 (27)	4 (13)	0.11

*PS* performance status, *CR* complete response, *OR* overall response, *PR*, partial response.

### Daunorubicin and cytarabine pharmacokinetics during FO infusion

The C_max_ of daunorubicin was measured at 92.2 µg/L (Q1; Q3: 44.8; 105.1), the AUC_∞_ at 414.9 ng/h, and the half-life (T_1/2_) at 3.5 h, in accordance with PK data from the literature (Table 4)^22–27^ Cytarabine clearance was highly variable with a mean of 1628 L/h.

**Table 4:** Pharmacokinetics of daunorubicin and cytarabine of the FAMYLY study compared to those of previous studies.

Study	N	Drug dosing	Inf. time	C_max_ (mg/L)	AUC (ng.h)	Cl (L/h)	T_1/2_ (h)
**Daunorubicin**							
^27^	4	1–1.5 mg/kg	45 min	475 ± NE	600 ± NE	NE	NE
^26^	70	60 mg/m^2^	60 min	200 ± 180	320 ± NE	270 ± 226	22.4 ± 15.4
^25^	12	50 mg/m^2^	15 min	210 ± 185	517 ± 296	221 ± 107	7.9 ± 3.0
^24^	21	45 mg/m^2^	IV bolus	NE	226 [31.6–570]	590 [90–2770]	1.5 [0.11–11.9]
^24^	24	50 mg/m^2^	60 min	105 ± NE	NE	129 ± 54	NE
FAMYLY	10	60 mg/m^2^	15 min	92.2 ± 82.9	415 ± 276	359 ± 15	3.5 ± 1.0
**Cytarabine**							
^22^		200 mg/m^2^/d	7d IVc	73.0 [12–266]	NE	135 ± 71	NE
FAMYLY		200 mg/m^2^/d	7d IVc	44.1 ± 69.8	72.1 ± 101.4	1 628 ± 1 672	10.7 ± 6.8

Numbers between brackets indicate [min–max] when the S.D. is absent.

*Inf* infusion, *IV* intravenous, *IVc* IV as a continuous infusion, *NE* not evaluated.

### Response

All 30 patients were evaluable for CR. Twenty-three achieved a CR after induction (77%; 95%CI [61; 92]), and 3 achieved a PR (10%). Fourteen patients (47%) received the protocol-specified second induction. The CR rate was not significantly different from the CR rate of 72% in the adverse cytogenetics subgroup of the daunorubicin arm of the LAM 2001 trial (*p* = 0.63). Propensity scoring adjustment for age differences or monosomal karyotype did not modify the CR rate.

### Survival

**Figure 3:**
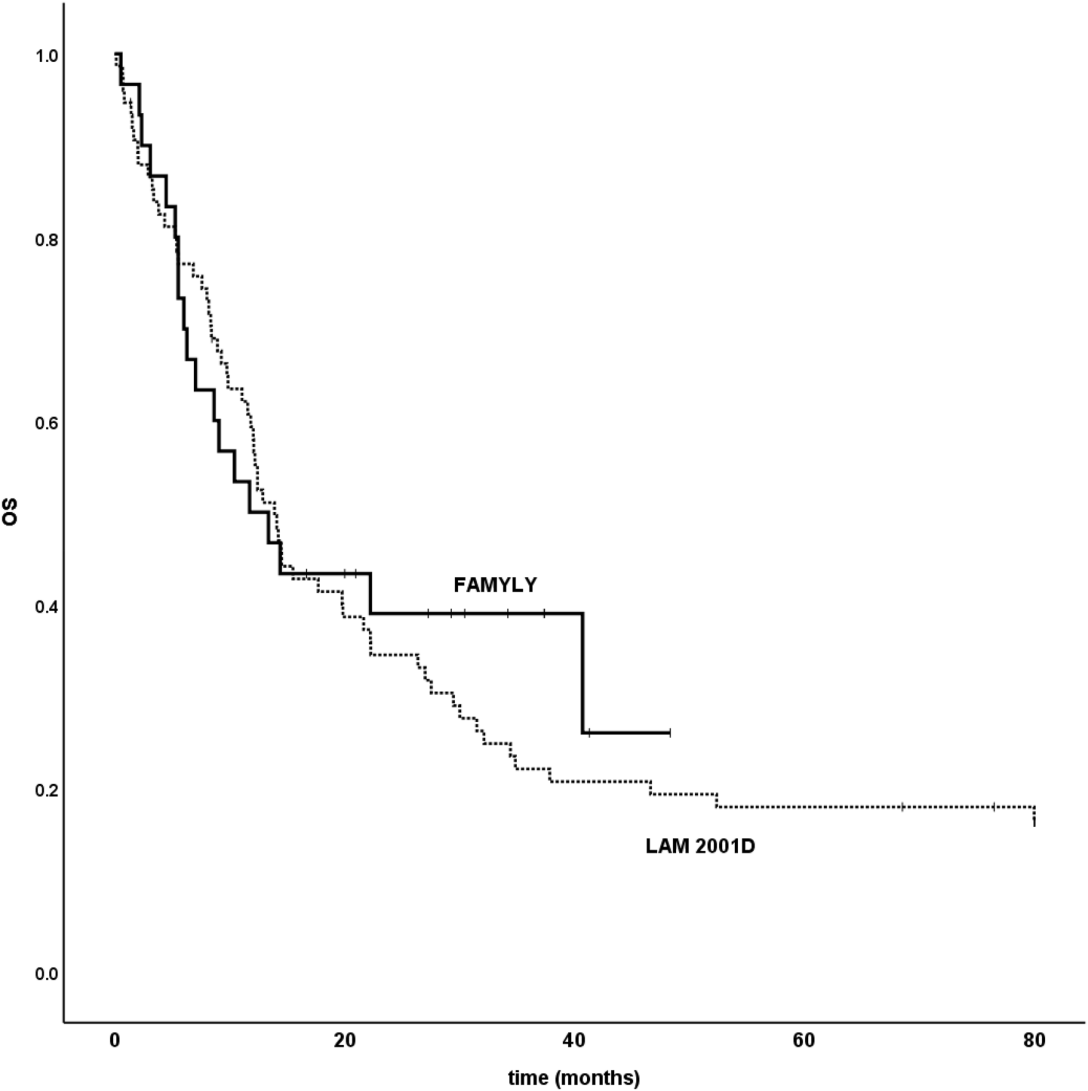
Historical comparison of survival data from the FAMYLY and LAM2001 trials. Overall survival (OS) was plotted from the inclusion date to the date of death or last follow up. The vertical marks indicate censored observations.

With a median follow-up of 12.9 months for the LAM2001 study, and 7.2 months for the FAMYLY study, median overall survival (not censored for allogeneic transplantation) was 13.9 months and 11.7 months, respectively, with no significant difference for the historical comparison (Fig. 3).

## Discussion

The results of the FAMYLY study show that the addition of IV FO to daunorubicin and cytarabine is feasible, with no unexpected toxicity. The pharmacokinetics of daunorubicin and cytarabine under FO infusion were consistent with those of previous reports in the literature, with high interindividual variability. We did not observe an in vivo effect of the FO emulsion alone on WBC count in any patient, as could have been expected based on previous ex vivo work^6^ nor an improvement in the CR rate for patients with high-risk AML. It is likely that the cell-killing effects observed in vitro in AML cell lines and primary blasts could not be reproduced in vivo because of insufficient access of PUFA to the bone marrow, even though plasma concentrations were close to those used in vitro. The novelty of our study is the demonstration that IV FO supplementation is feasible and transiently raises plasma DHA and EPA concentrations in patients with AML, and this may be useful for further applications. We also found that FO adjunction to 3 + 7 was associated with a higher proportion of grade 3 SAEs, associated with a lower proportion of grade 4 SAEs, when compared with the historical control data. However, the SAEs related to the study drug by investigator’s judgment was low. This difference between the two studies is likely to be a consequence of a more stringent declaration policy in the present phase 2 trial, as compared with the LAM 2001 phase 3 trial. In the literature, an enhanced toxicity of fish oil has not been observed^28^, rather, improved tolerability of cancer chemotherapy with the adjunction of oral PUFA has been reported in the setting of metastatic breast cancer supplemented with oral PUFA during chemotherapy^7^.

The strength of our study was the prospective evaluation of the effect of high-dose PUFA in humans with AML. One weakness of our study was the single arm design, with no 3 + 7 only comparator. This was a choice of the FILO group to launch a pilot study first and consider a comparative study if the early results of the pilot study were encouraging. As well, the two studies are 15 years apart and there have been changes in associated supportive care, and in adverse event declaration policies. This has complexified the comparison of tolerance, and does not help reach a definitive conclusion.

Overall, although EPA and DHA plasma enrichment is feasible via IV infusion of FO, we found no pharmacodynamic effect on WBC counts or oxidative stress enzyme expression and the CR rate was comparable to that of the historical study in the same high-risk AML population. It is possible that PUFAs may not have the same effect in vivo as in vitro because of the protective environment of the bone marrow niche^29^.

The landscape of AML therapy is quickly evolving, with new agents improving the response in molecular subgroups, such as *FLT3-ITD* or *FLT3-TKD* treated with midostaurin^30^ or IDH1/2-mutated AML treated with specific inhibitors^31,32^. Interestingly, treatment of secondary AML or AML with dysplastic features with CPX-351, a liposomal formulation of 3 + 7, improves patient outcome, probably because of improved tolerability^33^. In addition, combinations of low-intensity therapies, such as hypomethylating agents or low-dose cytarabine with promising candidates, such as venetoclax^34^ or glasdegib^35^ have shown promising results in the elderly AML population. These advances may soon be translated into the response and outcome of upfront AML therapy of younger patients. The question of whether or not the adjunction of FO formulations to modern therapies could help to improve the tolerability of emerging novel combinations, or in less aggressive hematologic malignancies, warrants further research.

## Conclusion

The adjunction of FO to 3 + 7 is feasible, and the complete response rate is not inferior to standard 3 + 7. These data do not support the adjunction of FO to 3 + 7.

## Supplementary Information

Supplementary Table S1.

## Data Availability

Supporting data is available on request from the authors.
