# Supplementary material for: Adjunction of a fish oil emulsion to cytarabine and daunorubicin induction chemotherapy in high-risk AML
Source: Sci Rep. 2022 Jun 13;12:9748. doi: 10.1038/s41598-022-13626-y (PMC9192636; doi:10.1038/s41598-022-13626-y)
Supplement: Supplementary file 1 — Supplementary Table S1. [file 41598_2022_13626_MOESM1_ESM.docx]

# Supplemental material

Supplemental Table 1. Primers used for RQ-PCR analysis.

| **Gene (isoform)** | **5’ primer** | **3’ primer** | **probe** |
| --- | --- | --- | --- |
| HMOX1 | ggcagagggtgatagaagagg | agctcctgcaactcctcaaa | catccagc |
| NQO1 | cggctttgaagaagaaaggat | cgcagggtccttcagtttac | tggtggag |
| SOD1 | gcatcatcaatttcgagcag | caggccttcagtcagtcctt | cttcccca |
| SOD2 | tccactgcaaggaacaacag | taagcgtgctcccacacat | ctgctggg |
| SOD3 | ctctcttttcaggagagaaagctc | aacacagtagcgccagcat | aggagctg |
| CAT | cgcagttcggttctccac | gggtcccgaactgtgtca | ctccagca |
| TXN | ttacagccgctcgtcaga | ggcttcctgaaaagcagtctt | ggctgctg |
| TXN2 | gagacaccagtggttgtgga | gcttggccaccatcttctc | ctggggcc |
| GLRX | ggcttctggaatttgtcgat | tgcatccgcctatacaatctt | cagccacc |
| GLRX2(1) | gtggcactcgctggaatc | cgtcgctaaattctccaaagat | ctccatcc |
| GLRX2(2) | gctggtttggagcaggag | ccaaagatgatgatgtattgctct | ggcggcgg |
| GLRX3 | tcctcaagaaccacgctgt | tgagaagatatcaaaactgctaaactg | tggtgga |
| GLRX5 | gtgataactggggcgttgtt | actcaggcatgcacagca | ctccagca |
| GPX1(1) | caaccagtttgggcatcag | gttcacctcgcacttctcg | ccaccacc |
| GPX1(2) | cccttgtttgtggttagaacg | gagagaagggcagctagaacc | ctcctcct |
| GPX2 | gtccttggcttcccttgc | tgttcaggatctcctcattctg | caggagaa |
| GPX3 | cagagatccttcctaccctcaa | ccctttctcaaagagctgga | aggtggag |
| GPX4 | tacggacccatggaggag | ccacacacttgtggagctagaa | ctgcccca |
| GPX7 | ccatcctgccttcaagtacc | ttccatctggggctactagg | ctccttcc |
| GSR | tgccagcttaggaataaccag | cctgcaccaacaatgacg | gctggaag |
| PRDX1(1,2,3) | cactgacaaacatggggaagt | tttgctcttttggacatcagg | ccagccag |
| PRDX2(1) | gccttccagtacacagacgag | gttgggcttaatcgtgtcact | cttcccca |
| PRDX2(3) | gcaactcagatgcaactctatctact | tgaactggagtttccatcttcat | cagcctcc |
| PRDX3(1,2) | ctggacaccggattctccta | gggtgatctactgatttaccttctg | ctgcttcc |
| PRDX4 | gcacctaagcaaagcgaaga | aaattctccatcgatcacagc | actgggaa |
| PRDX5(1,3) | tcctggctgatcccactg | atgccatcctgtaccaccat | ctccttcc |
| PRDX5(2) | cacccctggatgttccaa | ggacaccagcgaatcatctagt | ctccttcc |
| PRDX6 | caatagacagtgttgaggaccatc | tttctgtgggctcttcacaa | gctccagg |
| GAPDH | agccacatcgctcagacac | gcccaatacgaccaaatcc | cttcccca |
| ACTB | attggcaatgagcggttc | cgtggatgccacaggact | gctggaag |
| B2M | ttctggcctggaggctatc | tcaggaaatttgactttccattc | ccagccgc |
